# Suicide Research and Adolescent Suicide Trends in New Zealand

**DOI:** 10.1100/tsw.2008.46

**Published:** 2008-03-17

**Authors:** Said Shahtahmasebi

**Affiliations:** The Good Life Research Centre Trust, 975A South Eyre Road, RD 1, Rangiora, New Zealand

**Keywords:** holistic, suicide, health informatics, life events, New Zealand

## Abstract

In recent years, there have been a number of claims and counterclaims from suicide research using time series and longitudinal data; in particular, the linkage of increased antidepressant prescriptions to a decrease in suicide rates. Suicide time series appear to have a memory compounded with seasonal and cyclic effects. Failure to take into account these properties may lead to misleading conclusions, e.g., a downward blip is interpreted as the result of current knowledge and public health policies, while an upward blip is explained as suicide being complex depending on many variables requiring further research. In previous publications, I argued that this misuse of time series data is the result of an uncritical acceptance of a medical model that links mental ill-health to suicide. The consequences of such research behaviour are further increases in antidepressant prescriptions and medications to those who should not be prescribed them, with adverse effects showing across the population, e.g., the prescription of antidepressants to very young children (some under 1 year of age) in New Zealand. Moreover, the New Zealand Evidence-based Health Care Bulletin recommends an authoritarian approach for every interaction with a young person to check their psychosocial well-being. When viewed holistically, this kind of human behaviour makes researchers, policy makers (politicians), treatment, and practitioners, and society in general part of the problem rather than the solution. This paper explores some dynamic aspects of suicide, using only official data with particular reference to youth suicide, and suggests that the medical model of suicide is only an attempt to treat depression without addressing suicide, and recommends the creation of a unified database through understanding the society that individuals live in. It is hoped that this paper will stimulate debate and the collaboration of international experts regardless of their school of thought.

## INTRODUCTION

Adolescent suicide is a major public health concern globally. In some countries, it ranks between the first and third leading cause of death[1,2]. The suicide literature provides a historical, cultural, religious and philosophical background to suicide e.g. see [3,4]. For example, Ventegodt and Merrick[3] suggest that in some cultures suicide was viewed as a legitimate way out albeit to restore honour, as a rectification for mistakes or unforgivable acts, or a solution to difficult circumstances, e.g., to avoid committing other unforgivable acts. All major religions forbid suicide. In most western societies, suicide and suicidal thoughts and contemplation are viewed negatively and correlated to mental illness, thus emphasising the loss, waste, and negative impact on suicide survivors. However, Ventegodt and Merrick[3] rightly argue that life and death are real outcomes that provide real choices to think about, and are relevant to good living and very sound considerations. Indeed, making a choice is a process and processes are dynamic by nature, involving other social, economic, and environmental processes. Although all the major religions warn against suicide, these are presented as stern warnings with severe eternal consequences. However, at the end of the day, the choice is left to the individual "whoever commit suicide…" or "he who commits suicide…" will be punished in hell by the same method used for suicide. Therefore, as argued elsewhere[5,6], suicide is the outcome of a decision-making process. Making a decision is often governed by individual characteristics, and social, economic, and environmental processes[7].

In New Zealand, as in other countries, there has been a long-established view of the direction of causality from mental illness, specifically depression to suicide. This has been achieved by the hasty reporting of so-called research and the media's slant and uncritical reporting in particular. In announcing a $6.4 million campaign to reduce the impact of depression, the New Zealand Government claimed "We know that up to 90% of suicides are *caused* by depression and that each year 500 New Zealanders are dying by suicide." The Government further claimed "The World Health Organisation has predicted that by the year 2020, depression will be second only to cardiovascular disease, in contributing to the global burden of disease. We must tackle this problem head on and the National Depression Initiative will go a long way to achieving this objective." [http://www.beehive.govt.nz/ViewDocument.aspx?DocumentID=27352]. This announcement is a reflection of the ineffectiveness of health and social policies in reducing cardiovascular disease. However, a more conservative estimate of depression in the population suggests up to one in four women and one in ten men can expect to experience depression at some time in their lives [http://www.everybody.co.nz/page-75c9ff3f-7aa4-4b07-b63d-eaf5d92b88bb.aspx]. Applying these estimates to the New Zealand 2001 population estimates for males and females aged 15–85+ yields over 145,000 males and over 388,000 females who may be suffering from depression. If depression is such a strong causal factor, then should we not expect a much larger suicide mortality than the 500 quoted by the government? It is not surprising that when suicide occurs, the public mindset automatically blames depression/mental illness as the cause. The wisdom of the uncritical acceptance of this causal relationship was also challenged in earlier papers[5,6]. Within the suicide literature, despite the large volume of work, criticisms have been aired that our knowledge of suicide is based on scant scientific evidence[8,9,10]. The study design and the quality of data have been cited as being responsible for the ambiguities in the evidence. One of the consequences of accepting this relationship to explain suicide is to concentrate on building a case that would associate the suicide case with mental illness. Given the public mindset, it would be only too easy to suggest a link to mental illness after the event. For example, in suicide research, Beautrais[11,12,13] claims that depression and mental illness are the cause of suicide, Khan et al.[14] claim that antidepressants do not reduce suicide and may increase the risk of suicide, while Hall et al.[15] claim that antidepressants reduce suicide rates. The latter study[15] compared the first point in the time series with the last point, which happened to be lower than the first point and did not make much of the upward trend of suicide rates in between. Suicide data are — in particular those based on psychological, autopsy-type investigations such as those of Beautrais[11,12,13] — often plagued with a high degree of bias, the confounding and compounding of random effects such as error-in-variables with the structural error. Given the current public mindset that depression causes suicide, helped by the medical model and the media, the collection of data on suicide cases after the event from friends and relatives will be highly biased towards mental ill-health, as in the Canterbury Suicide Project[11,12,13]. These studies have failed to address the methodological issues related to design, data collection, and analysis, thus resulting in misleading conclusions. Furthermore, *The New Zealand Medical Journal* claims that depression is a common, serious, and significant illness and links it to suicide and recommends medication [http://www.nzma.org.nz/journal/117-1206/1200/]. It is not surprising to hear that young people have been prescribed antidepressants, including preschool children, but more alarming is the prescribing of antidepressants to some under 1 year old [http://www.nzherald.co.nz/section/1/story.cfm?c_id=1&objectid=10462684].

Viewed as an illness of the mind, suicide research has, over and above depression, followed many directions, such as a link with fatty acids[16,17], eating disorders and anorexia nervosa[18,19], persons with intellectual disability[20,21], cannabis and alcohol misuse[22,23,24,25], allergy symptoms[26], and clinical settings[24,27]; also see Shahtahmasebi[5] for more categories. Pharmacological treatment of mental illness, depression, and suicidal behaviour on its own may be controversial[28]; others suggest holistic medicine as a more effective treatment[29,30,31,32].

## BACKGROUND: SUICIDE AS TIME SERIES

Trend analysis of suicide mortality suggests cyclic (e.g., see Fig. 1) and seasonal effects[33]. These effects, if not accounted for, will make any association between antidepressants and a reduced mortality rate as proposed by Hall et al.[15] tenuous and questionable. However, the author is not aware of any studies in New Zealand that have taken into account the time series properties of suicide. Not surprisingly, a downward blip in the time series may easily be claimed as the result of current knowledge and policies. Conversely, an upward blip in the time series may be explained as suicide being very complex, which depends on many variables, and that more research is essential.

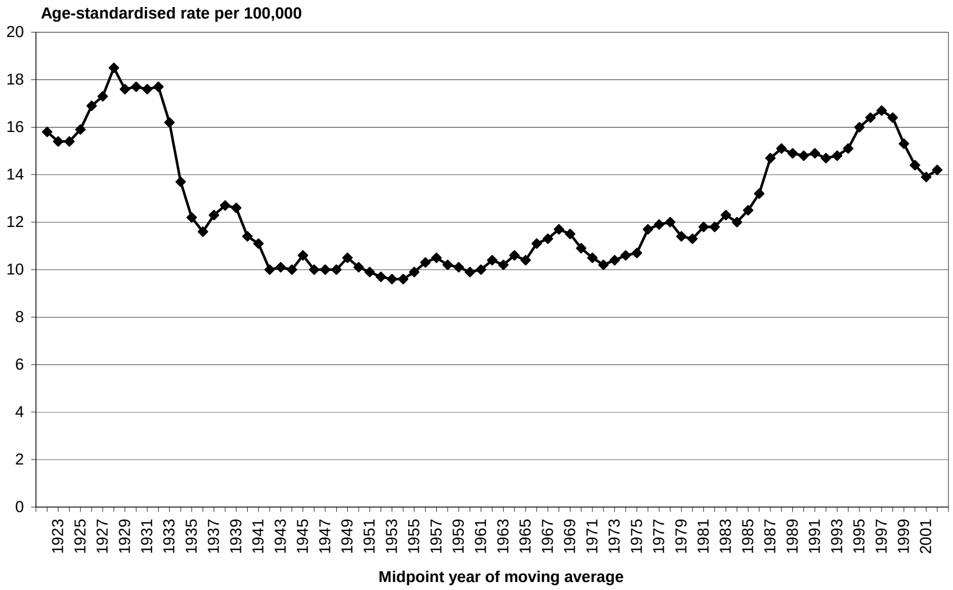

**FIGURE 1.** Age-standardised suicide rates, 3-year moving averages, 1921–2003. *Source*: New Zealand Health Information Service[34].

The major problem with suicide research is that the key informants are no longer available to provide an insight into the event. On the other hand, prospective designs require large samples over long periods of time, which are often ethically complex and financially prohibitive. For this reason, studies of suicide have mainly been retrospective, using psychiatric records and surveys of significant others (next of kin), or studying a subpopulation, e.g., those with a failed attempted suicide or those with a history of self-harm. Such studies are limiting as they are subjective and exclude the dynamics of life processes, and may lead to spurious relationships and give undue emphasis to some variables more than others. The view that suicide is not a result of mental illness is not new[35,36,37], but whatever the context (e.g., psychiatric, medical, social), suicide is a behavioural outcome and, therefore, compounded and confounded in suicide data will be high levels of measurement error and random noise due to subjectivity and unobserved/omitted characteristics associated with behavioural studies[38,39]. The main issue is, therefore, the availability of objective data to gain insight into the process of suicide in order to explore and examine its causal paths.

A common approach has been to present the sex/age and minority group conflicting trends for suicide, e.g., see Fig. 2. Such explorations have been used to target research and services or develop specialist preventional/interventional programmes. For example, in recent years, the authorities have claimed that youth suicide has a downward trend, but the suicide trend for some other groups, including Maori, is still upward. So the recommendations have been to learn from the success of the youth suicide prevention to affect suicide rates of other groups. Clearly, such an announcement of intentions will have serious social implications from research to government policy development on suicide. In this paper, I discuss youth suicide using only national data reported in official publications. The aim of this paper is to raise relevant issues concerned with suicide research, to stimulate debate and collaboration in order to develop a more coherent and holistic approach to investigating and understanding suicide.

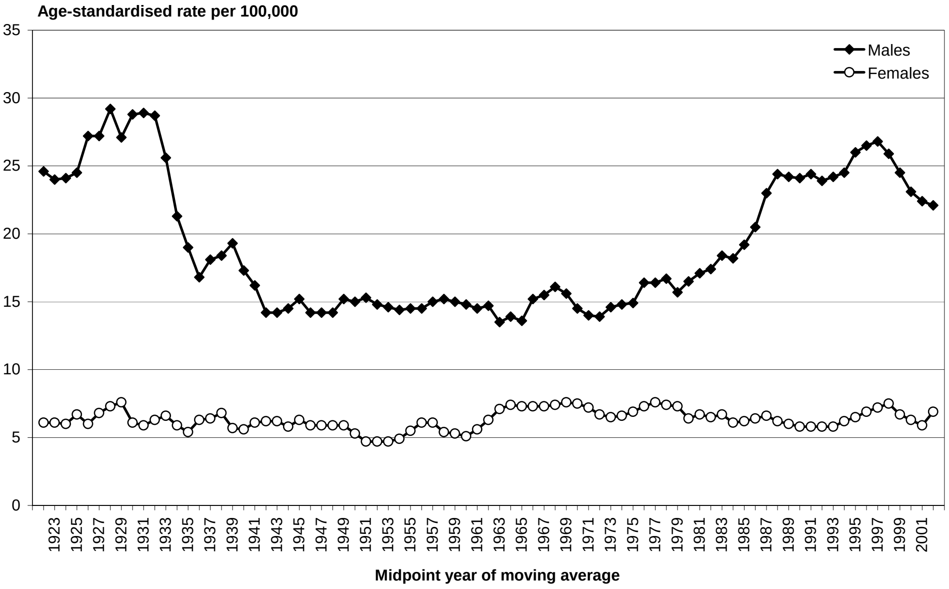

**FIGURE 2.** Death from suicide in New Zealand, by sex, 1921–2003. *Source*: New Zealand Health Information Service[34].

## PRESENTING SUICIDE AS A TIME SERIES

The introduction in a report from the New Zealand Ministry of Health[40] on suicide opens with “Suicide is a serious health issue that can be used as an indicator of mental health and wellbeing in the population.” From this opening sentence, there are two main points that reflect our understanding of suicide. The first, as suggested earlier, is a well-established assumption of a direct cause-and-effect relationship between mental illness and suicide. The second is the use of a fatal outcome as an *indicator* of a nonfatal outcome, e.g., mental illness!

Official reports produced by Public Health Intelligence Units or government departments often present the data graphically or in tabular form, and then describe the graphs or the tables (highs and lows in data). Very rarely do they go beyond what can be viewed to investigate the nature of the data and what they are trying to communicate[34,40]; hardly useful for informing the process of policy development.

It is interesting to note that although official reports appear to treat suicide as a time series, they often tend to restrict the time series to recent years where the last peak occurred. For example, reports and studies of suicide have frequently illustrated restricted or short-term trends such as that shown in Fig. 3[34]. It is a reasonable approach to take. A consistent downward trend over greater than 5 or 6 years may indicate a real change in the direction of the time series and may be attributed to public health policies. However, this interpretation may be valid if the time series has no memory, and seasonal and cyclic effects are not present. The danger is, of course, that these sequences of short-term trends are claimed as successful public policies and medical intervention while the complex nature of suicide has often been used as an explanation for the sequences of upward trends.

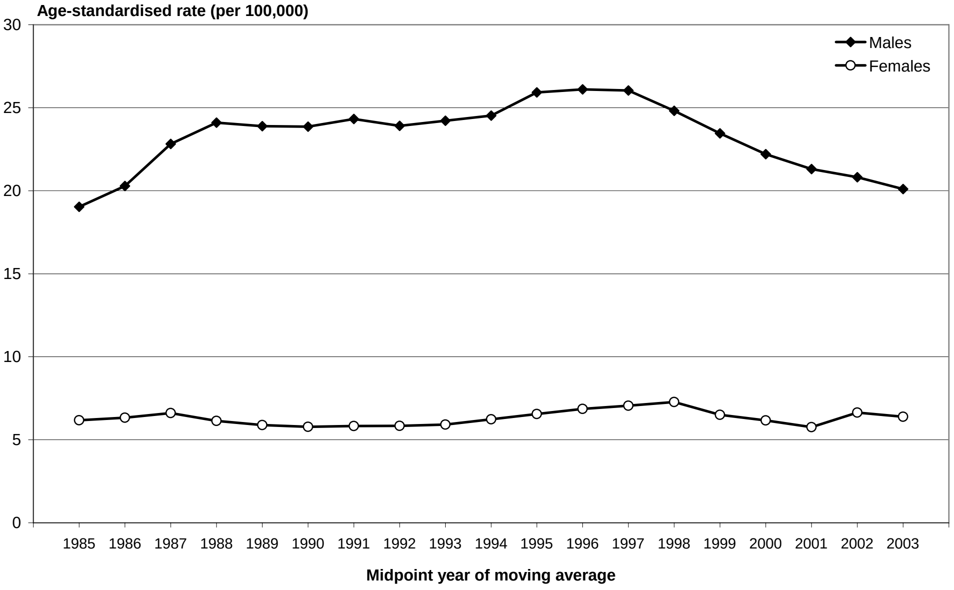

**FIGURE 3.** Suicide death rates, by sex, 3-year moving averages, 1984–2004. *Source*: New Zealand Health Information Service[34].

Similarly, a breakdown of suicide trends by age is shown in Fig. 4, and subsequent comments are restricted to the presentation of short-term suicide trends as shown in Fig. 5. It is plausible that due to this “short-termism”, attention has been shifted from addressing suicide holistically to a particular group(s) that may have been identified as at risk. This shift in emphasis may lead to a shift in resources from one group to another. Under such a reactionary policy development, the suicide rates and patterns control policy makers as opposed to policies bringing rates under control, leading to a see-saw effect. For the policy makers who are simply following peaks and troughs (leads and lags as in Fig. 4) in suicide, rates will regroup at the end of the cycle of one group and focus on the next group that has not quite completed the cycle. Thus, viewed in this way, it will not be difficult for the researchers and policy makers to become part of the problem rather than the solution.

## TRENDS IN ADOLESCENT SUICIDE

The suicide data for the 15–25 age group are presented in this section and shown in Fig. 6. Electronic data provided by the New Zealand Health Information Service only date back to 1992, thus limiting the trend to a short-term analysis. The main feature of the trends in Fig. 6 is that, overall, the female suicide trend appears fairly steady, while the male suicide rate appears to have a downward trend. However, even with

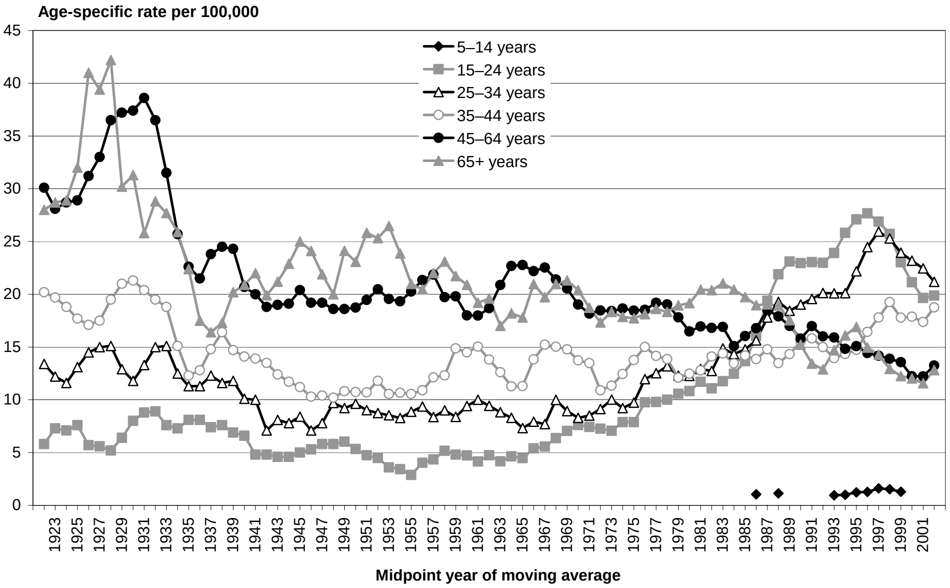

**FIGURE 4.** Suicide death rates, by age, 3-year moving averages, 1921–2003. *Source*: New Zealand Health Information Service[34].

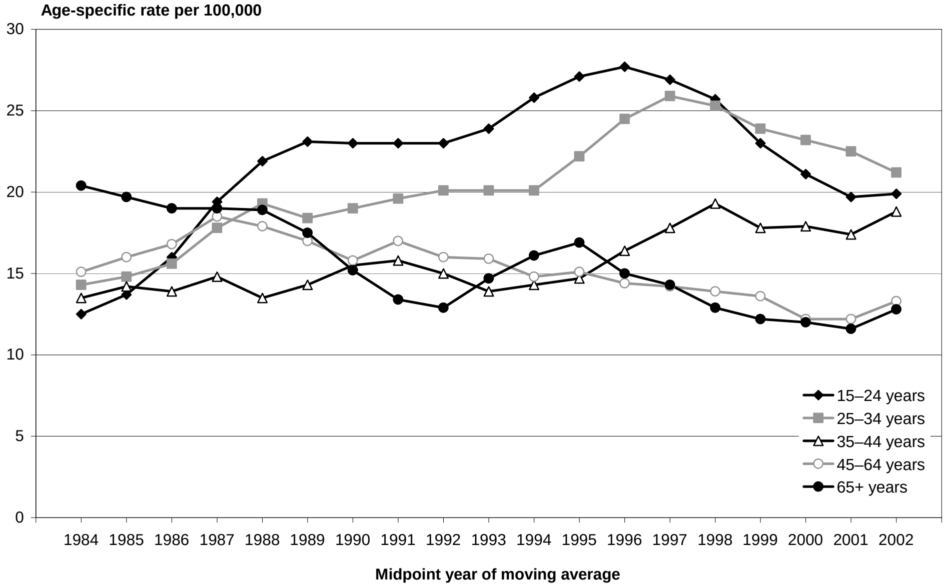

**FIGURE 5.** Suicide death rates, by age, 3-year moving averages, 1983–2003. *Source*: New Zealand Health Information Service[34].

the short-term trend, there are several features that merit further investigation. The first is the loose assumption, often made in the reporting and presentation of suicide data, that suicide rates may be similar for all those aged between 15 and 25. Some countries show a higher suicide rate for the late adolescence (20–25) than the mid adolescence (15–20), e.g., see Shek et al.[41]. The age category 15–25 is much more heterogeneous than 15–20 and 20–25, i.e., individuals aged 15 and 20 years may behave very differently to individuals aged 21 and 24, respectively. Thus, the classifying of suicide data into a 10-year class length

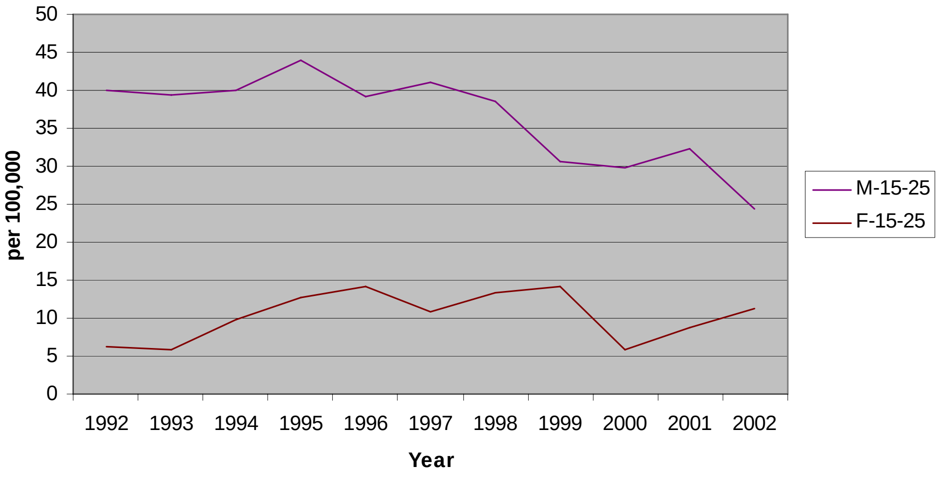

**FIGURE 6.** Youth suicide, age 15–25. *Source:* NZHIS.

lumps all individuals 15–25 years old into one group as though they were similar, which may misrepresent the suicide rates for mid and late adolescence. Accordingly, the data were reclassified to represent mid and late adolescence. We know that the female suicide rate is lower than that of males, so it would be interesting to compare trends between the age groups. In Figs. 7 and 8, mid adolescence is distinguished from late adolescence and. for comparison purposes. The 15–25 age group is also shown. It can be noticed that presenting data as a 10-year-wide category (15–25 years old) under-represents male suicide rate for late adolescence and over-represents it for mid adolescence (Fig. 7). Conversely, female suicide trends in Fig. 8 show that presenting adolescence as one 15–25 age category appears to over-represent suicides for the late adolescence and under-represents for the mid adolescence. By the same token, it is interesting to note from Fig. 8 that, unlike the male, the female suicide rate is higher in the 15–20 age group than the 20–25 age group. On the other hand, the trends appear to follow different dynamics: the male late adolescence trend appears to lead and the mid adolescence trend appears to lag, i.e., a peak in the 20–25 year olds coincides with a drop and is followed by a peak at the subsequent time points in the suicide trends of the 15–20 year olds. Conversely, the female mid adolescence trend leads the late adolescence suicide trend. The question to ask may be what environmental and economic factors govern these leads and lags? Why do peaks in the suicide rate for men in their late adolescence lead to peaks at the next time point in suicide rates for males in mid adolescence?

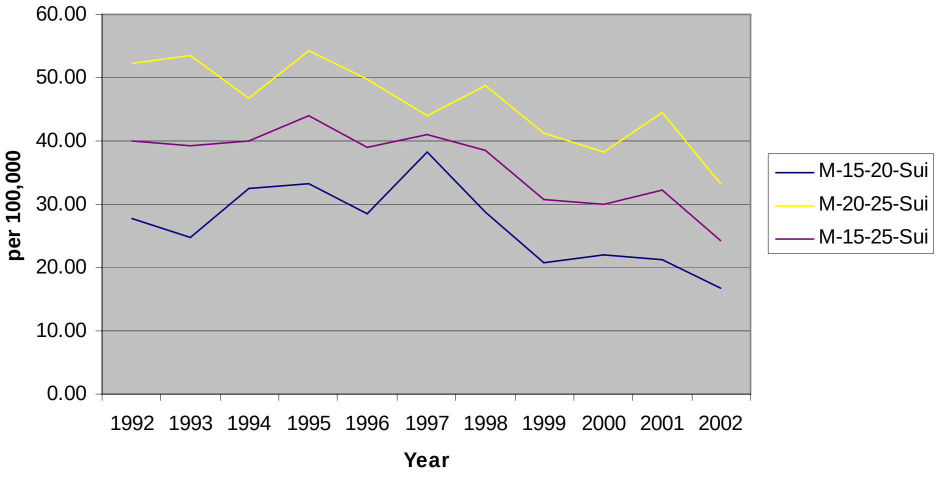

**FIGURE 7.** Youth suicide, males. *Source:* NZHIS.

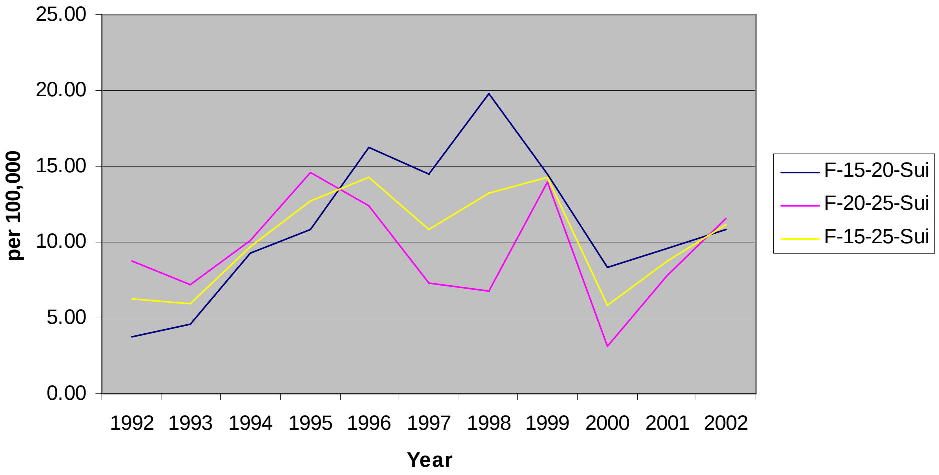

**FIGURE 8.** Youth suicide, females. *Source:* NZHIS.

The suicide mortality statistics are not known immediately and take time to be published. For each suspected suicide, there will have to be an inquest in the coroner's court before it is recorded as death by suicide or otherwise. Is there a "data collection" effect or could the pattern be explained by the increase in medication prescriptions? Thus, could there be a link to hospitalisation and treatment, i.e., is there a statistically significant treatment effect? However, previous studies suggest that over two-thirds of suicide cases have never had contact with psychiatric services. By the same token, could the pattern be explained by the number of attempters with the intention to die who received intervention, but then subsequently complete suicide?

The literature suggests that depression is underestimated in the general population[42] (also see http://www.beehive.govt.nz/ViewDocument.aspx?DocumentID=27352; http://www.everybody.co.nz/page-75c9ff3f-7aa4-4b07-b63d-eaf5d92b88bb.aspx; http://www.stuff.co.nz/4272340a12935.html), which means that quite a larger proportion of the public suffer depression unnoticed. If the medical model is correct, then we should observe quite a high suicide rate in the population. For instance, in a sample of the population of individuals with similar characteristics, one commits suicide while others do not. Therefore, the medical model of suicide is too simplistic a view of human behaviour; to gain insight into some of these questions, a more detailed study of suicide is long overdue. It is reasonable to assume that social, environmental, and other external processes are operating to reinforce an individual's beliefs and thought processes, or, to influence the decision-making process.

The literature often attributes self-harm and parasuicide as an important contributory factor to suicide[12,13]. As discussed elsewhere[5,6], about two-thirds of suicide cases have no hospital records, which makes this reported link somewhat questionable. Therefore, it would be interesting to explore the hospitalisation trends in conjunction with suicide rates, albeit using the available short-term time series. Time series shown in Figs. 7 and 8 are presented again in Figs. 9 and 10 to include hospitalisation rates.

It is clear that the hospitalisation rates due to self-harm are much higher than suicide rates. However, similar to suicide trends, females in their mid adolescence show a higher hospitalisation rate than those in their late adolescence; conversely, the 20- to 25-year-old males show a higher hospitalisation rate than those in mid adolescence (15–20 years old). It is interesting to note that the hospitalisation trends for late adolescence for both sexes appear fairly flat while the rate for 15- to 20-year-old males shows a downward trend. Once again, in the absence of historical data over a longer period, it is difficult to envisage the direction and slope of the 10-year trends shown in Figs. 9 and10. A comparison of hospitalisation (Figs. 9 and10) and suicide rates (Figs. 7 and 8) between the sexes and age groups does not appear to show any pattern, although there may be a weak presence of lag and lead between the suicide and hospitalisation rates, respectively. However, the trends are too short to substantiate it visually.

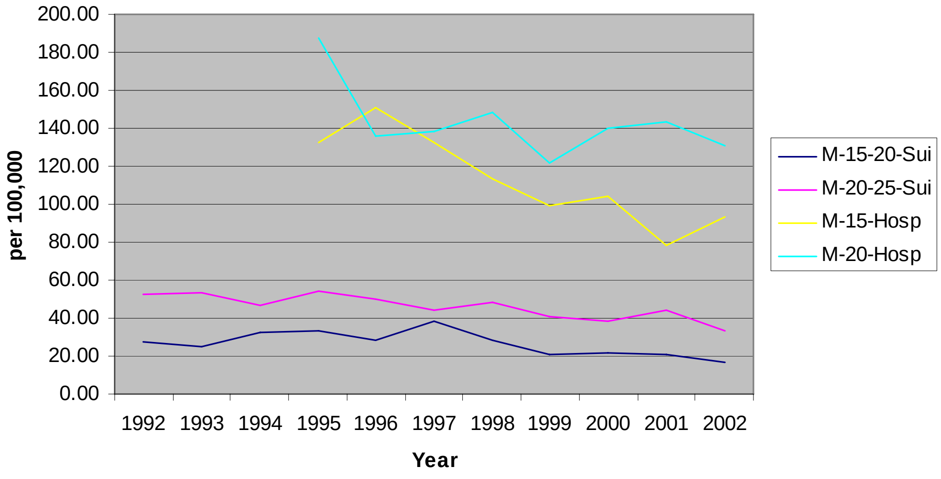

**FIGURE 9.** Suicide and hospitalisation, males. *Source:* NZHIS.

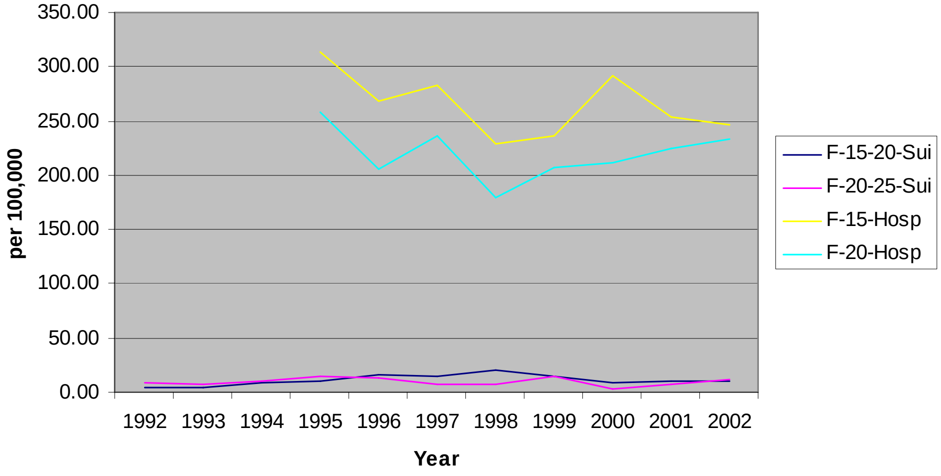

**FIGURE 10.** Suicide and hospitalisation, females. *Source:* NZHIS.

## DISCUSSION

It is possible that hospitalisation rate is a more reasonable indicator of depression in the population than suicide. There is, however, some evidence to support the view that self-harm or parasuicide has a different distribution to that of suicide[43,44,45]. There is no doubt that there will be an overlap between the two populations, perhaps due to a suicide attempt going fatally wrong, or conversely, a failed first attempt. This overlap may also explain the weak appearance of a lag and lead in the suicide and hospitalisation rates. Clearly, the hospitalisation rates may not be taken as an indicator of suicide rates and the two populations are different.

In its suicide prevention strategy document[46] launched in 2006 the New Zealand Government claimed that after eight years male suicide is at its lowest now and credited this to its youth suicide strategy. Over the same period, New Zealand has enjoyed reasonable economic success. In its overview of New Zealand economy (http://www.treasury.govt.nz/economy/overview/2007/04.htm; also see http://www.treasury.govt.nz/publications/media-speeches/speeches/growth-collaborative), the treasury claims that since 1998 the economy has performed strongly with the exception of a short weaker period during 2001

when growth slowed to around 2%, much lower than the 5.7% peak reached in the year to June 2000. However, the economy regained momentum, with a combination of two good agricultural seasons, relatively high world prices for New Zealand's export commodities, a low exchange rate and a robust labour market contributing to strong income flows throughout the economy. During the period 2002 to 2004, growth in the GDP was generally in the 3.5% to 4.5% range peaking just above this at 4.6% annual average growth in December 2002. This period of strong growth included a period in which the economy was subject to several negative temporary events. These included travel disruptions and uncertainty due to the conflict in Iraq, the outbreak of Severe Acute Respiratory Syndrome (SARS), and the effects of dry weather on hydro-electricity production and farm output. Recently the Government announced a structural surplus and signalled a direct cut to income tax (http://www.newstalkzb.co.nz/newsdetail1.asp?storyID=126929; also see http://www.scoop.co.nz/stories/PA0711/S00104.htm). All these "positive" economic activities may lead to growth at the micro- or household level. As commented in the treasury's summary report (http://www.treasury.govt.nz/economy/overview/2007/04.htm), the main sources of recent growth have been household spending and business investment. Furthermore, the recent strength of household spending has been supported by relatively low interest rates, the strong labour market and rising house prices.

It is noteworthy that the downturn in economy in 2001 appears to have coincided with an upturn in suicide rates for most age groups in 2002, see Fig. 5. The report, however, forecasts a slowdown in migration and housing, and increasing interest rates in order to slow down the escalating house prices. These changes in the economy will affect domestic growth in spending despite the promise of a direct cut in income tax.

Is it, therefore, too simplistic and naïve of the authorities to present a short-term downward suicide trend that has coincided with an upbeat economy and claim it to be the results of suicide prevention initiatives and policies? Any downturn in suicide trends is a success and it is the desire of all to see a continuous drop in suicide. However, the question must be asked: is it possible that what is being observed in New Zealand is a cycle effect rather than (or compounded) by any treatment/prevention effects? Indeed, there is some evidence to suggest some effects on suicide rates from macroeconomic trends[47,48,49,50,51,52]. Clearly, macroeconomic changes do not directly cause suicide; their effects on suicide rates would be over time through cumulative inertia. In other words, the relationship is over time through individuals', social, and economic parameters, such as employment rates, disposable income (e.g., higher house prices and higher interest rates leading to the largest single monthly outgoing on mortgage repayment), and possibly divorce.

How do all these macrolevel activities affect youth suicide? A quick re-examination of Fig. 4 suggests that apart from the 65+ age group, all the other groups appear to follow a similar pattern, albeit at different rates. This indicates that youth suicide rates may be subject to influence from the same processes that influences those age groups active in the economy. Although external processes, such as the economy, may not directly affect the youth in the same way that it may affect the older age groups, it may affect the young person's future life perspective through education, social, and interaction with other processes.

On the whole, Fig. 4 reflects a societal effect on suicide rates. During a recession or economic downturn, negative perceptions are often magnified. The magnitude of the effects will clearly depend upon both the individual and society's tolerance and resilience to coping and addressing problems.

The case of a mid adolescent who had committed suicide has been briefly reported elsewhere[6]. At the inquest, it was heard that there was a rumour that the young person was being picked on at school and that 3 months before his death, he had changed his eating habits. Although no further details were provided as to how the diet had changed, there is some evidence to suggest a relationship between diet and suicide[16,17,18,19,53,54]. It is interesting to note that both families and health professionals in their search for answers had focused on the case's mental health even though there was no evidence of mental illness. For example, in giving evidence to the coroner, the young person's GP had said: "I am desperately sad we had no insight into his mental health problems and so were not able to prevent this tragedy." Why should the GP automatically assume "mental health problems" about a case who was reported to have been a happy and popular person with no obvious health problems and no evidence of mental ill-health? On the other hand, it is not clear how many potential suicide cases are actually cured or prevented. What about those cases that the mental health services had insight into and the individuals still committed suicide, as in the following case from Australia?

In recent media coverage of a celebrity suicide case in Australia (http://www.stuff.co.nz/4262547a1860.html), several points could be underlined; the case was on antidepressant medication (although we have no knowledge of compliance at this stage), the case appeared happy, and was making plans for the future. After the event (suicide), the psychiatrist suggested that cases with deep depression are good at hiding their feelings and intentions! So even when there is insight into one's mental health status, after the event, a higher level of mental illness is implied! On the contrary, the anecdotal evidence from personal files suggests that the choice for life or death is made *not* in a state of mental confusion, but during clarity of mind.

In another case, an individual suffering from depression made attempts to self-harm [personal communication]. The person was aware of her/his actions and frequently chose methods of low to medium risk. For example, during one of her/his recent spells, s/he drove into a power line in a rural area. S/he has now set up a self-help group to help others deal with depression.

Ventegodt and Merrick[3,32] present the treatment of a 22-year-old patient presenting with a long history of childhood sexual abuse, incest, and rape. In this case, the authors adopted their "holistic" approach to developing treatments and applied it to this case. After 100 h of intensive therapy, the case was supported and taught to confront her life issues, understand them, and deal with them in such a way as to help her make an informed and "free" choice about "life" or "death", as opposed to a "command" choice based on drug therapy. The authors quite rightly argue against the current medical model of treating suicide; the use of medication and drugs may only treat the symptoms, and the real issues that the patient should be made aware of and given the skills to deal with them are masked.

The main point of concern that all of us (not just the practitioners and researchers) must remember is that at the centre of this decision-making process is a person. In the case of the young adolescent, the individual has already gone through their own process of decision making, by which time it is too late for any external influence/intervention as these would have been interpreted as interference. Although these suicide cases may appear as sudden decisions, nevertheless, they suggest that the individual is in the driving seat and is in control. In the case of the individual with episodes of depression, self-harm may be his/her way of breaking out of the depression, but not necessarily by dying, even though death may be one possible outcome. In some cases, self-harm may coincide with the case's desire to seek help. In this case, the individual does not wish to die, but uses self-harm for whatever reason, but as mentioned above s/he chose "life" by setting up a self-help group. This is in contrast to the Australian individual who chose "death". Again, it is the individuals who are in the driving seat and making a decision to seek help and direction or not. In the final example, even though a holistic approach was used, the person had shown a degree of willingness to agree to a treatment. Regardless of whether the person had suicidal intentions, it was her decision processes that led her to receive treatment. The apparent success of the treatment rests solely with the "holistic" approach of placing the patients in the driving seat and providing them with a useful map and the skills to navigate through past life events and to prepare for the future (e.g., also see Cassidy et al.[55]).

It must be noted, as reported earlier, that most of the suicide cases had no psychiatric records, suggesting that, for these people, the decision-making process had been completed and executed before any "intervention" could be actioned. Clearly, in these cases, not even a holistic treatment would have been effective because of the choice they made. However, such an approach may be modified and extended to understand individuals through an understanding of the society they live in, in order to influence the individual's process of decision making. In medical treatments, a "holistic" approach is often referred to as treating the person and not just the physical or emotional symptoms. Ventegodt and Merrick[3] suggest that we must, as holistic physicians and health practitioners, support and guide our patients through the *dynamics* of suicide. To understand the dynamics of human behaviour in the current age of an advanced information culture, the "holistic" notion must go beyond the individual and accept that decision-making processes are influenced by many other processes, such as social, economic, environment, media[7]. Physicians on their own have little or no control over these dynamic processes.

The Good Life Approach[7] may be adopted to develop social and health policy actions incorporating possible influences from these processes. As shown in Fig. 11, the Good Life assumes the individual as well as society as the central processes that are subject to influence from other processes. Some of these processes we know about and are related to human behaviour; politics, economy, education, public health, and medicine, while others such as the environment are inter-related with human behaviour, but we do not have complete control over them. The problem is that the dynamics of human behaviour may influence these processes in a conflicting and diverging direction to contradict and lead to adverse interactions between the processes. Erroneous results and misconclusions from a biased and subjective assessment of suicide will have further ramifications for policy and treatment. For example, we may question the effects of antismoking or antiviolence policies where, ignoring the behaviour of the politicians, the media's portrayal of a hero is a violent smoker who dabbles in risky behaviour.

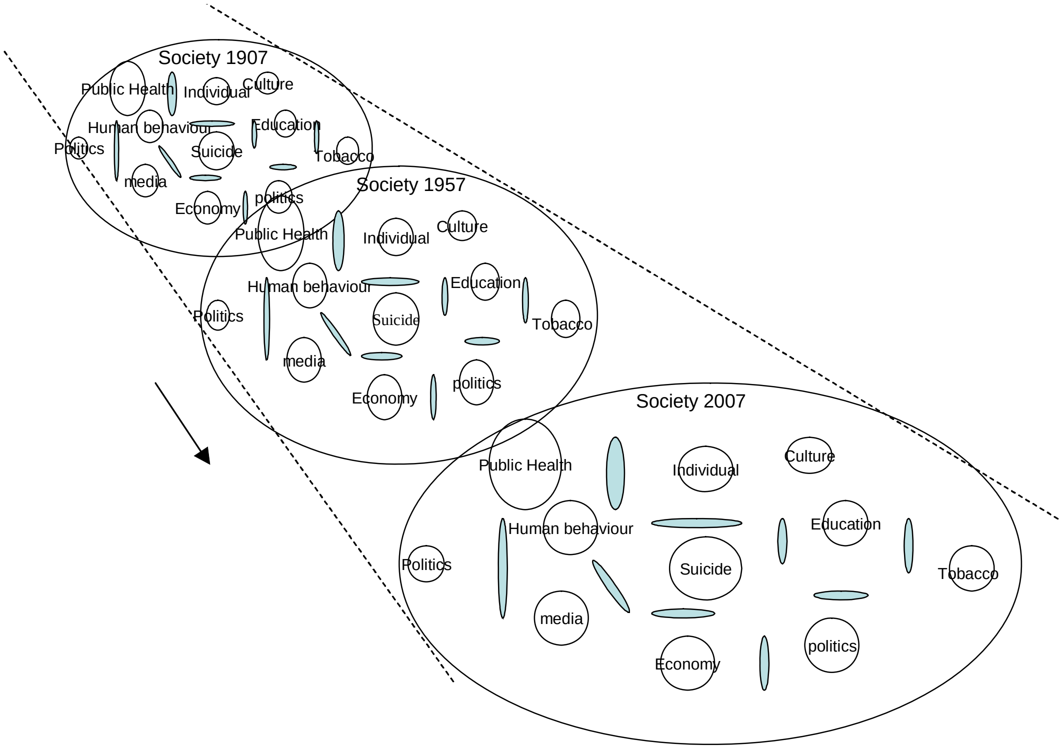

**FIGURE 11**. An example of a conceptual holistic model of suicide.

Another example may be, as discussed earlier, the significant increase in prescription of antidepressants over recent years, and more worrying is the age range and particularly the very young who have become consumers of antidepressants. This is equivalent to the manipulation of the outcome in order to get the appearance of a desired outcome; i.e., throwing the baby out with the bathwater!

Furthermore, the role that religion plays in society, at least at an individual level, has been underestimated. The religious warnings and threats of eternal punishment by the same method used to take one's own life are one of many ways of stabilising the thought processes and hence the process of decision making. The literature provides evidence that religion may have a positive effect on well-being and a negative effect on suicide rates[56,57,58,59] and that spiritualism should not be ignored from treatment[57,60]. Again, it is difficult to ascertain the net effect of an individual's religious beliefs on decision process outcomes in the presence of other social, education, economic, and environmental processes. However, in terms of suicide, a comparison of various cultures suggests that Muslim countries and certain cultures that may have built-in protective factors appear to have the lowest suicide rates[58,59].

The Good Life approach is to take "holistic" further, to mean the development of subprocesses (the oblong solid shapes in Fig. 11), to harmonise other processes to counteract the conflict and divergence between all processes. In this case, it will mean placing the individual or society at the centre and in the driving seat as *opposed* to the notion of finding the magic pill that is the answer to every social ill from behaviour to ill health.

## CONCLUSION

The uncritical acceptance of the depression-suicide relationship means that treatment is hardly questioned, especially one that restricts and takes control away from the individuals using medications and health policies. As a result, suicide and depression are lumped together as one outcome and both appear to receive the same treatment. Thus, the treatment effects are ignored. In other words, treatment of individuals with suicidal intention for depression may have adverse effects on their mental well-being, which may aggravate the circumstances that had merited suicide as a choice in the first place. This is not too dissimilar to the example of elderly people who refuse institutional medical treatments outside of their home because of the fear of being kept under control or sent to residential care homes away from their own home; they often wish to die in their own home[61,62]. The way society has been dealing with some health issues has created a fear in the social subgroups to the extent that they will not ask for help, for doing so may result in their loss of control[62]. Indeed, it was shocking to see The New Zealand Evidence-based Health Care Bulletin report on an authoritarian youth development approach in clinical practice (http://www.nzgg.org.nz/newsletter/dsp_article_template.cfm?articleID=454). It suggests that young people are at high risk of mental disorders, and it recommended that every interaction with a young person should be used as an opportunity to check their psychosocial well-being regardless of the presenting complaint. It further recommends the use of a clinical assessment tool. It is horrifying to imagine that a visit to a clinician/practitioner may turn out to be something completely different. It would not be surprising if this kind of treatment behaviour becomes part of the problem as the fear of seeking treatment may lead to the young individual's refusal to take up treatment**.** On the other hand, the current uncritical acceptance of the depression-suicide relationship has paved the way for the unnecessary increase in prescribed antidepressant medication across all age groups and an authoritarian approach to prevention and treatment. The main issue here is the basing of any results and conclusions on biased and subjective assessment of suicide; public perception of suicide and its causes that will become even more emphasised after the event (suicide).

One way of gathering objective life event histories is to use data mining techniques to extract data from all health and community records for all suicide cases and not just those with a psychiatric record[5,6]. Such an exercise requires major funding and support. Indeed, as reported[5,6], similar studies have been carried out in Europe and Australia. However, if appropriately designed together with an appropriate methodology for analyses, such projects could lead to the creation of a unified database that may be used for the development of appropriate service planning and, if necessary, early intervention.

Regardless of an economic downturn, unemployment, sex, age, divorce, depression or mental ill-health, and other popular social and environmental factors that have been associated as the cause of suicide, the fact remains that in a population stratified by all these factors, a small proportion make a death over life decision while others choose life. Therefore, the question is what governs the process of decision making that a downturn in the national economy triggers a "negative" choice for some and for others the trigger may be divorce or an episode of depression that may have been influenced by macroeconomic changes. Are these random life events proxy for the same and possibly an unobserved effect leading to suicide?

We must treat suicide for suicide and not for depression using more suitable holistic approaches such as that proposed by Ventegodt and Merrick[3]. However, in terms of researching suicide, it is time for appropriately designed, good-quality research to gain a real insight into suicide as a behavioural process outcome. However, presently, it is wise to take a step back and take a self-critical look at our knowledge and understanding of suicide, and accept suicide as the outcome of a decision-making process most likely influenced by other processes, such as socio- and environmental processes, processes are dynamic by nature; e.g., see Kral[63].

## References

1. Mann, J.J. (2005) Foreword. In *Suicidal Behaviour in Adolescence: An International Perspective*. Merrick, J. and Zalsman, G., Eds. Freund Publishing, Tel Aviv. pp. 1–2.
2. Merrick, J. and Zalsman, G. (2005) Suicide from an international perspective. *TheScientificWorldJOURNAL* **5,** 888–890.
3. Ventegodt, S. and Merrick, J. (2005) Philosophy of life and suicide: to be or not to be is still the question. In . In *Suicidal Behaviour in Adolescence: An International Perspective*. Merrick, J. and Zalsman, G., Eds. Freund Publishing, Tel Aviv. pp. 9–17.
4. Maharajh, H.D. and Abdool, P.S. (2005) Cultural aspects of suicide. *TheScientificWorldJOURNAL* **5,** 736–746.
5. Shahtahmasebi, S. (2003) Suicides by mentally ill people. *TheScientificWorldJOURNAL* **3,** 684–693.
6. Shahtahmasebi, S. (2005) Suicide in New Zealand. *TheScientificWorldJOURNAL* **5,** 527–534.
7. Shahtahmasebi, S. (2006) The good life: a holistic approach to the health of the population. *TheScientificWorldJOURNAL* **6,** 2117–2132.
8. De Leo, D. (2002) Why are we not getting any closer to preventing suicide? *Br. J. Psychiatry* **181,** 372–374.
9. Institute of Medicine. (2002) Reducing Suicide: A National Imperative. *National Academic Press,* Washington DC.
10. Cutcliffe, J.R. (2003) Research endeavours into suicide: a need to shift the emphasis. *Br. J. Nurs.* **12(2)**, 92–99.
11. Beautrais, A.L., Joyce, P.R., and Mulder, R.T. (1994) The Canterbury suicide project: aims, overview and progress. *Community Ment. Health N. Z.* **8(2),** 32–39.
12. Beautrais, A.L. (2001) Suicides and serious suicide attempts: two populations or one? *Psychol. Med.* **31,** 837–845.
13. Beautrais, A.L. (1996) Serious Suicide Attempts in Young People: A Case Control Study. [PhD]. Christchurch School of Medicine.
14. Khan, A., Warner, H.A., and Brown, W.A. (2000) Symptom reduction and suicide risk in patients treated with placebo in antidepressant clinical trials: an analysis of the Food and Drug Administration database. *Arch. Gen. Psychiatry* **57,** 311–317.
15. Hall, W.D., Mant, A., Mitchell, P.B., Rendle, V.A., Hickie, I.B., and McManus, P. (2003) Association between antidepressant prescribing and suicide in Australia, 1991-2000: trend analysis. *BMJ* **326(7397),** 1008.
16. Brunner, J., Parhofer, K.G., Schwandt, P., and Bronisch, T. (2002) Cholesterol, essential fatty acids, and suicide. *Pharmacopsychiatry* **35(1),** 1–5.
17. Ainiyet, J. and Rybakowski, J. (1996) Low concentration level of total serum cholesterol as a risk factor for suicidal and aggressive behavior. *Psychiatr. Pol.* **30(3),** 499–509.
18. Dancyger, I.F. and Fornari, V.M. (2005) A review of eating disorders and suicide risk in adolescence. *TheScientificWorldJOURNAL* **5,** 803–811.
19. Latzer, Y. and Hochdorf, Z. (2005) A review of suicidal behaviour in anorexia nervosa. *TheScientificWorldJOURNAL* **5,** 820–827.
20. Merrick, J., Merrick, E., Lunski, Y., and Kendel, I. (2005) Suicide behaviour in persons with intellectual disability. *TheScientificWorldJOURNAL* **5,** 729–735.
21. Merrick, J., Merrick, E., Morad, M., and Kendel, I. (2005) Adolescents with intellectual disability and suicidal behaviour. *TheScientificWorldJOURNAL* **5,** 724–728.
22. Maharajh, H.D. and Konings, M. (2005) Cannabis and suicidal behaviour among adolescents: a pilot study from Trinidad. *TheScientificWorldJOURNAL* **5,** 576–585.
23. Sher, L. (2006) Risk and protective factors for suicide in patients with alcoholism. *TheScientificWorldJOURNAL* **6,** 1405–1411.
24. Sher, L. (2006) Alcohol and suicide: neurobiological and clinical aspects. *TheScientificWorldJOURNAL* **6,** 700–706.
25. Sher, L. (2006) Relation between rates of geriatric suicide and consumption of alcohol beverages in European countries. *TheScientificWorldJOURNAL* **6,** 383–387.
26. Postolache, T.T., Lapidus, M., Sander, E.R., Langenberg, P.H., Robert, G., et al. (2007) Changes in allergy symptoms and depression scores are positively correlated in patients with recurrent mood disorders exposed to seasonal peaks in aeroallergens. *TheScientificWorldJOURNAL* **7,** 1968–1977.
27. Pompili, M., Girardi, P., Lester, D., and Tatarelli, R. (2006) Suicide soon after generalized tonic-clonic seizure. A possible peri-ictal phenomenon? *TheScientificWorldJOURNAL* **6,** 356–360.
28. Farley, R.L. (2005) Pharmacological treatment of major depressive disorder in adolescents. *TheScientificWorldJOURNAL* **5,** 420–426.
29. Ventegodt, S., Kandel, I., Neikrug, S., and Merrick, J. (2005) Clinical holistic medicine: the existential crisis--life crisis, stress, and burnout. *TheScientificWorldJOURNAL* **5,** 300–312.
30. Ventegodt, S., Andersen, N.J., Neikrug, S., Kandel, I., and Merrick, J. (2005) Clinical holistic medicine: holistc treatment of mental disorders. *TheScientificWorldJOURNAL* **5,** 427–445.
31. Ventegodt, S. and Merrick, J. (2005) Suicide from a holistic point of view. *TheScientificWorldJOURNAL* **5**, 958–766.
32. Ventegodt, S., Clausen, B., and Merrick, J. (2006) Clinical holistic medicine: the case story of Anna. III. Rehabilitation of philosophy of life during holistic existential therapy for childhood sexual abuse. *TheScientificWorldJOURNAL* **6,** 2080–2091.
33. Hakko, H., Rasanen, P., Tiihonen, J., and Nieminen, P. (2002) Use of statistical techniques in studies of suicide seasonality, 1970 to 1997. *Suicide Life Threat. Behav.* **32(2),** 191–208.
34. Ministry of Health (2006) New Zealand Suicide Trends: Mortality 1921–2003, hospitalisations for intentional self-harm 1978–2004. Monitoring Report No 10. Ministry of Health, Wellington, New Zealand.
35. Maris, R.W., Berman, A.L., and Silverman, M.M. (2000) *Comprehensive Textbook of Suicidology*. Guilford Press, New York.
36. Maris, R.W. (1981) *Pathways to Suicide: A Survey of Self- Destructive Behaviours*. Johns Hopkins University Press, Baltimore.
37. Wasserman, D. (2001) *Suicide: An Unnecessary Death*. Martin Dunitz, London.
38. Shahtahmasebi, S. and Berridge, D. (2005) Teenage smoking: a longitudinal analysis. *Int. J. Adolesc. Med. Health* **17(2),** 137–155.
39. Shahtahmasebi, S. (2004) Quality of life: a longitudinal analysis of correlates of morale in old age. *TheScientificWorldJOURNAL* **4,** 100–110.
40. Ministry of Health (2006) Suicide Facts: 2004–2005 data. Ministry of Health, Wellington, New Zealand.
41. Shek, D.T.L., Lee, B.M., and Chow, J. (2005) Adolescent suicide in Hong Kong: 1980 to 2003. In *Suicidal Behaviour in Adolescence: An International Perspective*. Merrick, J. and Zalsman, G., Eds. Freund Publishing, Tel Aviv. pp. 235–264.
42. Blair-West, G.W., Mellsop, G.W., and Eyeson-Annan, M.L. (1997) Down-rating lifetime suicide risk in major depression. *Acta Psychiatr. Scand.* **95(3)**, 259–263.
43. Pattisson, E.M. and Kahan, J. (1983) The deliberate self-harm syndrome. *Am. J. Psychiatry* **107,** 867–872.
44. Simeon, D., Stein, J.D., and Holander, E. (1998) Depersonalization disorder and self-injurious behaviour. *J. Clin. Psychiatry* **56(Suppl 4),** 36–39.
45. Favazza, A.R. (1998) The coming of age of self-mutilation. *J. Nerv. Ment. Dis.* **186,** 250–268.
46. Associate Minister of Health (2006) The New Zealand Suicide Prevention Strategy 2006-2016. Ministry of Health 2006. Wellington. http://www.moh.govt.nz/suicideprevention.
47. Yang, B. (1992) The economy and suicide: a time-series study of the USA. *Am. J. Econ. Sociol.* **51(1),** 87–99.
48. Granados, J.A.T. (2005) Increasing mortality during the expansions of the US economy, 1900–1996. *Int. J. Epidemiol.* **34(6),** 1194–1202.
49. Kposowa, A.J. (2001) Unemployment and suicide: a cohort analysis of social factors predicting suicide in the US National Longitudinal Mortality Study. *Psychol. Med.* **31(1),** 127–138.
50. Dooley, D., Catalano, R., Rook, K., and Serxner, S. (1989) Economic stress and suicide: multilevel analyses. Part 2: Cross-level analyses of economic stress and suicidal ideation. *Suicide Life Threat. Behav.* **19(4),** 337–351.
51. Brainerd, E. (2001) Economic reform and mortality in the former Soviet Union: a study of the suicide epidemic in the 1990s. *Eur. Econ. Rev.* **45(4–6),** 1007–1019.
52. Weyerer, S. and Wiedenmann, A. (1995) Economic factors and the rates of suicide in Germany between 1881 and 1989. *Psychol. Rep.* **76(3 Pt 2),** 1331–1341.
53. Dancyger, I.M. and Fonari, V.M. (2005) Eating disorders and suicide: is there a relationship? In *Suicidal Behaviour in Adolescence: An International Perspective*. Merrick, J. and Zalsman, G., Eds. Freund Publishing, Tel Aviv. pp. 143–155.
54. Latzer, Y. and Hochdorf, Z. (2005) Dying to live: attachment to life in anorexia nervosa. In *Suicidal Behaviour in Adolescence: An International Perspective*. Merrick, J. and Zalsman, G., Eds. Freund Publishing, Tel Aviv. pp. 157–167.
55. Cassidy, B., Clarke, A., and Shahtahmasebi, S. (2004) Quality of life: information and learning resources in supporting people with severe life-changing injuries to return to independence. *TheScientificWorldJOURNAL* **4,** 536–543.
56. Ventegodt, S., Merrick, J., and Andersen, N.J. (2003) Quality of life as medicine: a pilot study of patients with chronic illness and pain. *TheScientificWorldJOURNAL* **3,** 520–532.
57. Webb, D. (2005) Bridging the spiritual gap. *Australian e-Journal for the Advancement of Mental Health (AeJAMH)* **4(1)**: http://www.auseinet.com/journal/vol4iss1/webb.pdf .
58. Maharajh, H.D. and Abdool, P.S. (2005) Culture and suicide. In *Suicidal Behaviour in Adolescence: An International Perspective*. Merrick, J. and Zalsman, G., Eds. Freund Publishing, Tel Aviv. pp. 19–32.
59. Morad, M., Merrick, E., Schwarz, A., and Merrick, J. (2005) A review of suicide behaviour among Arab adolescents. *TheScientificWorldJOURNAL* **5,** 674–679.
60. Birnbaum, L. and Birnbaum, A. (2004) In search of inner wisdom: guided mindfulness mediation in the context of suicide. *TheScientificWorldJOURNAL* **4,** 216–227.
61. Flanagan, J. and Holmes, S. (1999) Facing the issue of dependence: some implications from the literature for the hospice and hospice nurses. *J. Adv. Nurs.* **29(3),** 592–599.
62. Feinberg, A.W. (1997) The care of dying patients. *Ann. Intern. Med.* **126(2),** 164–165.
63. Kral, M.J. (1994) Suicide as social logic. *Suicide Life Threat. Behav.* **24(3),** 245–255.
